# Treatment of Iron Deficiency With Intravenous Ferric Carboxymaltose in General Practice: A Retrospective Database Study

**DOI:** 10.14740/jocmr1974w

**Published:** 2014-10-16

**Authors:** Martina Kuster, Damian N. Meli

**Affiliations:** a Institute of General Practice, Universtity of Bern, Switzerland

**Keywords:** Iron deficiency, Ferric carboxymaltose, General practice

## Abstract

**Background:**

Iron deficiency is a frequent problem in general practice. Oral supplementation may in some cases not be well tolerated or not be efficient. Intravenous ferric carboxymaltose may be an alternative for iron supplementation in general practice. The aim of the present study was to analyze the indications for and the efficacy of intravenous ferric carboxymaltose in a primary care center.

**Methods:**

We retropectively analyzed electronic data from 173 patients given intravenous ferric carboxymaltose between 2011 and 2013 in primary care center with 18 GPs in Bern, Switzerland.

**Results:**

Of all patients, 34% were treated intravenously due to an inappropriate increase in ferritin levels after oral therapy, 24% had side effects from oral treatment, 10% were treated intravenously due to the patients explicit wish, and in 39% of all cases, no obvious reason of intravenous instead of oral treatment could be found. Intravenous ferric carboxymaltose led to a significant increase in hemoglobin and serum ferritin levels. Side effects of intravenous treatment were found in 2% of all cases.

**Conclusion:**

We conclude that treatment with intravenous ferric carboxymaltose is an efficient alternative for patients with iron deficiency in general practice, when oral products are not well tolarated or effective. As treatment with iron carboxymaltose is more expensive and potentially dangerous due to side effects, the indication should be placed with (more) care.

## Introduction

Iron deficiency is the most common nutritional deficiency worldwide which can affect people of all ages from small children [1] to the elderly [2]. Symptoms of iron deficiency can vary grossly from distriction in self-helping [1] to chronic fatigue, lack of concentration, anginal pain, lightheadedness, swelling of ankles, breathlessness, pallor, dizziness, palpitations, headache, irritability or restless legs syndrome [3, 4].

In general practice, chronic fatigue due to iron deficiancy is a frequent problem and patients are often prescribed oral iron supplementation products [5]. However, oral iron treatment can cause gastrointenstinal side effects, such as acid reflux, constipation, diarrhea, nausea or flatulence, and sometimes, treatment is not effective [6]. In 2007, ferric carboxymaltose was approved in Switzerland for intravenous treatment of iron deficiency [7]. A dose of up to 1,000 mg of ferric carboxymaltose can be administrated intravenously within 15 min [8]. The total iron concentration in the serum increases rapidly in a dose-dependent manner after administration of ferric carboxymaltose. Ferric carboxymaltose is rapidly cleared from the circulation and is distributed primarily to the bone marrow (about 80%) but also to the liver and spleen [8].

In the present study, we retrospecively analyzed the indication, the efficacy and the safety of intravenous ferric carboxymaltose in a primary care center in Bern.

## Methods

We retrospectively analyzed patients from electronic medical records from one primary care center with 18 GPs in Bern, Switzerland. We searched for all patients who were given ferric carboxymaltose (Ferinject^®^) from January 01, 2011, until June 30, 2013. Only patients who had a full blood count (FBC) and recorded ferritin levels before and up to 8 months after administration were included in our study. For this retrospective anonymous database analysis, according to the Swiss law [9] and the local ethical committee, no ethical approval was needed for this study. Data were analyzed using GraphPad Prizm^®^ statistical software. A P value of < 0.05 was considered to be statistically significant.

## Results

A total of 422 patients between January 01, 2011 and June 30, 2013 received therapy with intravenous ferric carboxymaltose. Of those 422 patients, 249 were excluded; a total of 173 patients fullfilled the chosen criteria.

**Figure 1:**
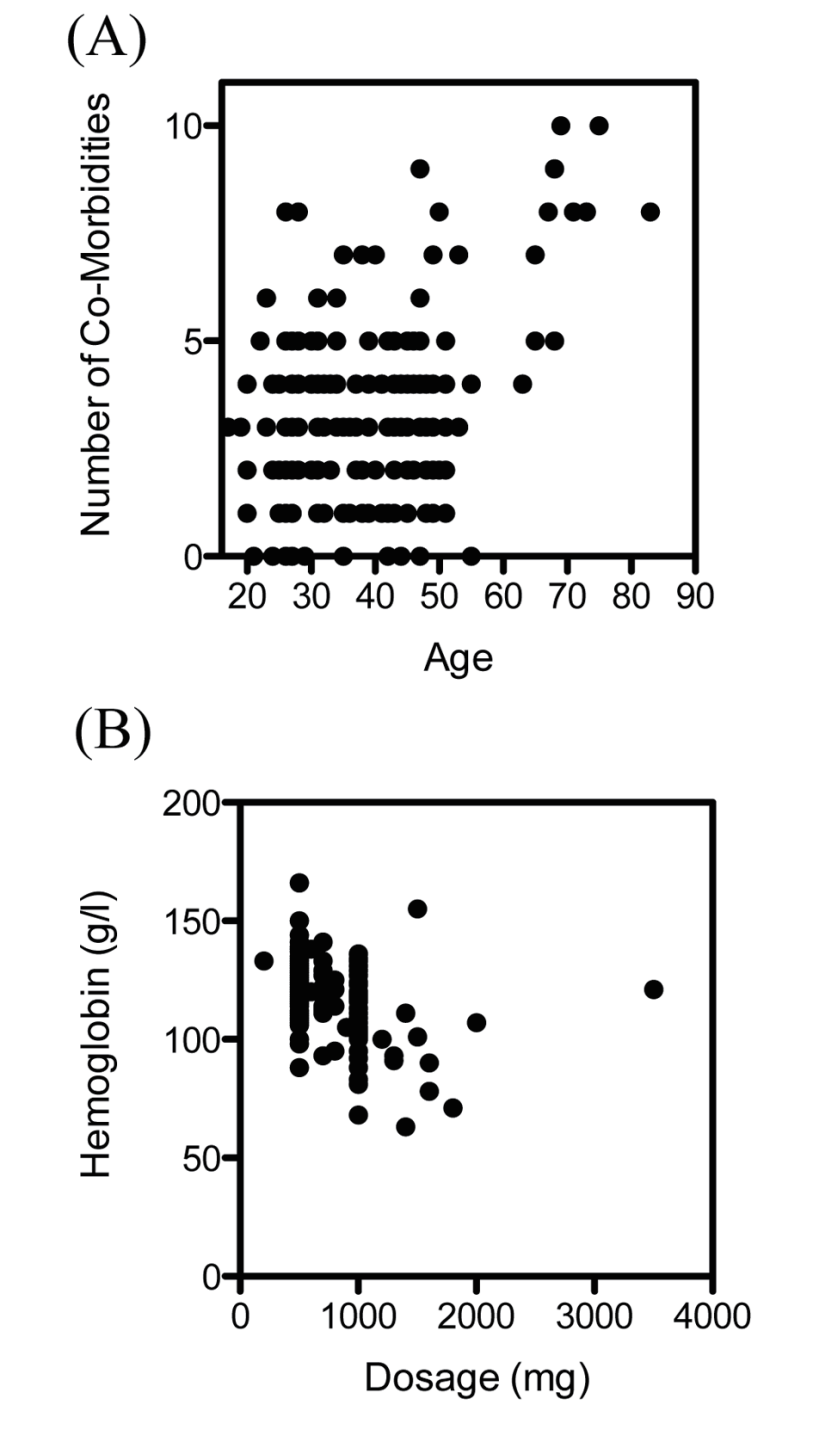
Age, comorbidities, initial hemoglobin and dosage of ferric carboxymaltose. (A) Number of comorbidities and age of patients. Each dot represents one patient. The number of comorbidities correlated significantly with the patients age (r = 0.26, P < 0.001). (B) Initial hemoglobin and dosage of ferric carboxymaltose given. Each dot represents one patient. The dosage correlated negatively with the initial hemoglobin (r = -0.45, P < 0.001).

The patient characteristics, symptoms and reasons for intravenous treatment are summarized in Table 1. Most of the patients who received intravenous iron were female (163 of 173 patients, 94.2%). The mean age of female patients was 33, and the mean age of male patients was 49. The mean number of comorbidities was 3.3 in female patients and 4.9 in male patients. The number of comorbidities correlated significantly with the patients age (r = 0.26, P < 0.001) (Fig. 1A). The symptoms found in the patients notes varied from chronic fatigue, hair loss, brittle fingernails, exercise intolerance, palpitations, irritability, dizziness, burning tongue sensation, anginal pain, tremor and sleep disorders to lack of concentration.

**Table 1:** Age of Patients Who Were Given Intravenous Ferric Carboxymaltose, the Number of Their Comorbidities, Symptoms of Iron Deficiency and Reasons for Intravenous Treatment

	Women	Men
Number of patients	163	10
Mean age (years)	39	52
Number of comorbidities	3.3	4.9
Symptoms		
Fatigue	111	7
Depression	3	
Hair loss	12	
Splitting nails	6	
Exercise intolerance	54	3
Other	78	6
Reasons for intravenous administration		
Patients wish	17 (10.4%)	0
Side effects from tablets	39 (23.9%)	3 (30%)
Unsatisfactory effect	56 (34.4%)	2 (20%)
Other	63 (38.7%)	4 (40%)

Fifty-eight patients (34%) were treated intravenously instead of orally due to an inappropriate increase in ferritin levels after oral therapy; 42 patients (24%) had side effects from oral treatment. Seventeen patients (10%) requested intravenous treatment due to upcoming holidays, stress, not being able to swallow pills, or the avoidance of side effects. Three patients (2%) were treated intravenously because of severe symptomatic anemia. In 67 patients (39%), no obvious reason could be found for the administration of intravenous instead of oral treatment.

**Figure 2:**
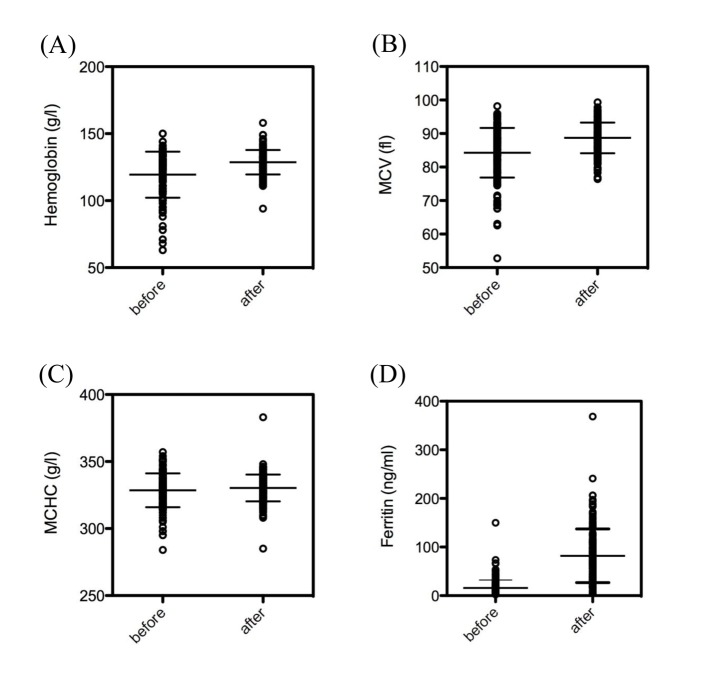
Effect of intravenous ferric carboxymaltose on hemoglobin, MCV, MCHC and ferrritin. (A) Ferric carboxymaltose led to a significant increase of hemoglobin (119.4 ± 1.3 vs. 128.7 ± 0.7 g/L, N = 173, P < 0.0001). (B) MCV was also significantly higher after administration of ferric carboxymaltose (84.3 ± 0.6 vs. 88.7 ± 0.3 fL, N = 173, P < 0.0001). (C) There was no difference between MCHC before and after ferric carboxymaltose (328.5 ± 1.0 vs. 330 ± 0.8 g/L, N = 173, ns). (D) Treatment with ferric carboxymaltose led to significant higher ferritin levels (15.9 ± 1.2 vs. 82.1 ± 4.2 ng/mL, N = 173, P < 0.0001). The data show the mean ± SEM.

The dosage of intravenous ferric carboxymaltose given was dependent on the initial levels of hemoglobin (Fig. 1B), as suggested in treatment recommendations. Intravenous ferric carboxymaltose administration led to a significant increase of hemoglobin (119.4 ± 1.3 vs. 128.7 ± 0.7 g/L, N = 173, P < 0.0001) (Fig. 2A). Mean corpuscular volume (MCV) was also significantly higher after administration of ferric carboxymaltose (84.3 ± 0.6 vs. 88.7 ± 0.3 fL, N = 173, P < 0.0001) (Fig. 2B). There was no difference between mean corpuscular hemoglobin concentration (MCHC) before and after ferric carboxymaltose (328.5 ± 1.0 vs. 330 ± 0.8 g/L, N = 173, ns) (Fig. 2C). Treatment with ferric carboxymaltose led to significantly higher ferritin levels (15.9 ± 1.2 vs. 82.1 ± 4.2 ng/mL, N = 173, P < 0.0001) (Fig. 2D). Allergic reactions to the intravenous ferric carboxymaltose were seen in three cases (2%); paravenous infusion with colored skin never occurred (data not shown).

## Discussion

We found that patients treated with intravenous carboxymaltose in this particular primary care center were mainly female. Female patients were also younger and healthier than their male counterparts, which is not surprising, due to menstruation as a frequent cause of iron deficiency. However, patients of all ages and morbidities were given intravenous ferric carboxymaltose, and the treatment was efficient and safe. Side effects were rare, and their rate was comparable to other studies [8]. Intravenous therapy should preferrably be given to patients who experience intolerable side effects after oral treatment, and in cases of malcompliance, repeatedly menorrhagia or gastrointestinal bleeding, chronic inflammatory bowel disease, postoperative bleeding, anemia with severe cardiac disease and dialysis [8]. Intravenous treament is more expensive than oral treamtent, and health insurance policies in Switzerland cover intravenous ferric carboxymaltose only after unsuccesful oral treatment [7]. However, in our study, intravenous carboxymaltose was also given for other reasons, such as patients’ own preferences. In addition, it was also given to patients with normal hemoglobin and subnormal ferritin levels, where the evidence of a benefit of intravenous iron treatment is weak [10]. In line with a recent warning by the pharmacovigilance of the Swiss Agency for the Authorization and Supervision of Therapeutic Products (Swiss medic) about potential fatal side effects of intravenous ferric carboxymaltose [11], we believe that the indication of an intravenous ferric carboxymaltose treament should be placed with more care.
